# Deep learning-based network pharmacology for exploring the mechanism of licorice for the treatment of COVID-19

**DOI:** 10.1038/s41598-023-31380-7

**Published:** 2023-04-10

**Authors:** Yu Fu, Yangyue Fang, Shuai Gong, Tao Xue, Peng Wang, Li She, Jianping Huang

**Affiliations:** Alibaba Business School, Hangzhou Normal University, Hangzhou, 310000 China

**Keywords:** Neurological models, Bioinformatics

## Abstract

Licorice, a traditional Chinese medicine, has been widely used for the treatment of COVID-19, but all active compounds and corresponding targets are still not clear. Therefore, this study proposed a deep learning-based network pharmacology approach to identify more potential active compounds and targets of licorice. 4 compounds (quercetin, naringenin, liquiritigenin, and licoisoflavanone), 2 targets (SYK and JAK2) and the relevant pathways (P53, cAMP, and NF-kB) were predicted, which were confirmed by previous studies to be associated with SARS-CoV-2-infection. In addition, 2 new active compounds (glabrone and vestitol) and 2 new targets (PTEN and MAP3K8) were further validated by molecular docking and molecular dynamics simulations (simultaneous molecular dynamics), as well as the results showed that these active compounds bound well to COVID-19 related targets, including the main protease (Mpro), the spike protein (S-protein) and the angiotensin-converting enzyme 2 (ACE2). Overall, in this study, glabrone and vestitol from licorice were found to inhibit viral replication by inhibiting the activation of Mpro, S-protein and ACE2; related compounds in licorice may reduce the inflammatory response and inhibit apoptosis by acting on PTEN and MAP3K8. Therefore, licorice has been proposed as an effective candidate for the treatment of COVID-19 through PTEN, MAP3K8, Mpro, S-protein and ACE2.

## Introduction

Coronavirus disease 2019 (COVID-19) has had a significant impact on global health systems and economic development due to its highly infectious nature and complex pathogenesis^1^. Existing therapies, including conventional treatments (e.g., oxygen therapy) and immunomodulators, can only play a preventive role, and the rapid development of specific drugs and vaccines targeting COVID-19 has become the greatest challenge^2^. Studies have shown that traditional Chinese medicine (TCM) can improve clinical symptoms, delay disease progression, as well as reduce mortality and recurrence rates in patients with COVID-19^3,4^. Of the available formulas, preventive prescriptions, and therapeutic prescriptions for confirmed cases proposed by TCM, licorice is one of the most frequently used for the treatment of COVID-19^5,6^.

Licorice is a perennial herb commonly used in TCM^7^, leguminous or plant rhizomes and dried roots are frequently used in medicinal preparations^8^. Many licorice compounds and corresponding targets have been shown to play a central role in the treatment of COVID-19 through network pharmacology analysis, as well as in in vivo or in vitro studies^9–11^. In terms of compounds, for example, Glycyrrhizic Acid, and other compounds found in licorice can bind to Mpro, ACE2 and S proteins, respectively, which could inhibit COVID-19 replication and block virus binding sites^12–14^; in terms of targets, MAPKs, ILs and NF-kB can regulate the MAPK signaling pathway, the IL-17 signaling pathway and the NF-kB signaling pathway, exerting anti-inflammatory and immunomodulatory effects^13^.

**Figure 1:**
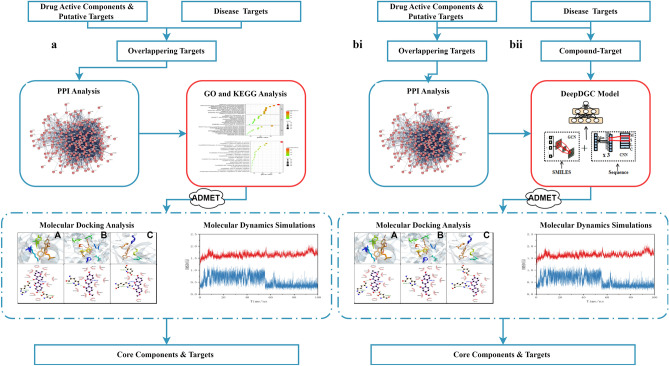
(**a**) Flow chart of traditional network pharmacology process (P1); (**b**) Improved flow chart for two processes: (i) process P2 and (ii) process P3.

Traditional network pharmacology, namely P1 (Fig. 1a) in this study, an research method is often used to identify active compounds and targets^15^, has the advantages of being comprehensive, systematic, and holistic, which is consistent with the multi-compound, multi-target, and multi-pathway characteristics of TCM, expanding the potential applications of TCM research^16^. However, it still presents some challenges, such as the lack of comprehensive data on various drugs, genes, and proteins^17^. Therefore, we proposed a deep learning (DL)-based network pharmacology method, in which a model integrated with a drug-target interaction (DTI) method was adopted [Highlight the innovative nature of the method].

Based on above new method, two new processes were proposed, namely P2 (Fig. 1bi) and P3 (Fig. 1bii). (1) For process P2, since the traditional network pharmacology approach does not consider characteristic information about the molecular structure of proposed drugs, integrating GO and KEGG enrichment analysis in process P1 with the DTI prediction, it is possible to predict more compounds and targets. (2) Compared to process P2, which identifies some important compounds and targets after PPI analysis, process P3 takes into account all the active compounds of licorice and COVID-19 targets and performs a prediction of DTI, making full use of all available information.

In fact DTI is one of the most direct and effective methods for discovering active compounds and targets. Several new DTI methods have been developed in recent years, all of which have shown promising results, for example, DeepDTA, a model only extracts the sequence information of targets and compounds by the convolutional neural network (CNN)^18^. And DeepPurpose, which combined the current optimal models, achieved better results compared to the previous model^19^. However, these models only considered a single feature of compound information. Therefore, we proposed a new model called DeepDrugTargetInteractionandGraphConvolutional.

(DeepDGC) for DTI prediction, which included two DL algorithms, graph convolutional neural network (GCN) and CNN, to extract more characteristic information from the molecular structure of the compound. GCN and CNN were used to obtain two representations of the compound—the molecular map and the Morgan fingerprint, respectively. In addition, CNN was used to learn the amino acid sequence of the disease targets. After two vectors were generated in the above two steps, they were inserted into a fully connected layer, followed by a regression layer, in which the output was the compound-target affinity value.

According to the above description, this study included a total of three processes, i.e., P1 (Fig. 1a), P2 (Fig. 1bi), and P3 (Fig. 1bii). Here, P1 was the traditional network pharmacology process; P2 and P3 were the improved processes proposed in this study.

## Materials and methods

### Acquisition of active compounds and licorice targets

We retrieved 249 compounds by searching the TCMSP^20^ databases with the keyword "licorice". After screening the criteria of oral bioavailability (OB) ≥ 30% and drug-likeness (DL) ≥ 0.18, 92 active compounds remained. The corresponding targets of the active compounds were obtained from the TCMSP^20^, SwissTargetPrediction^21^, PharmMapper^22^ and GeneCards^23^ databases, and 1140 targets were named after deleting duplicate items.

### Acquisition of disease targets

Searching the GeneCards^23^, OMIM^24^, DrugBank^25^ and other databases with the keywords "COVID-19" produced 13,542 targets for COVID-19 after deleting duplicate values.

### Acquisition of overlapping targets

A total of 774 overlapping targets remained after intersection screening using Venny^26^ from 1140 licorice targets and 13,542 targets for COVID-19, which were considered potential targets of licorice acting in COVID-19.

### Acquisition of key compounds and key targets by analysis of PPI networks

The overlapping targets were loaded into the STRING database^27^ to obtain the PPI network. After the removal of isolated targets, the PPI network was imported into Cytoscape software^28^, where the Centiscape plug-in was used to screen for the key targets, with three parameters (Degree unDir, Betweenness unDir and Closeness unDir) used as thresholds. The key compounds were then acquired according to the key targets.

### Acquisition of core compounds and core targets by GO and KEGG enrichment analysis

Based on the key targets, the Metascape database^29^ was used to conduct GO and KEGG enrichment analysis (*p* < 0.01), and a licorice-compound-target-pathway network was built to screen core targets by the Cytoscape software. All compounds corresponding to the core targets on the network were defined as core compounds.

### Predictions of compounds and targets based on DeepDGC

**Figure 2:**
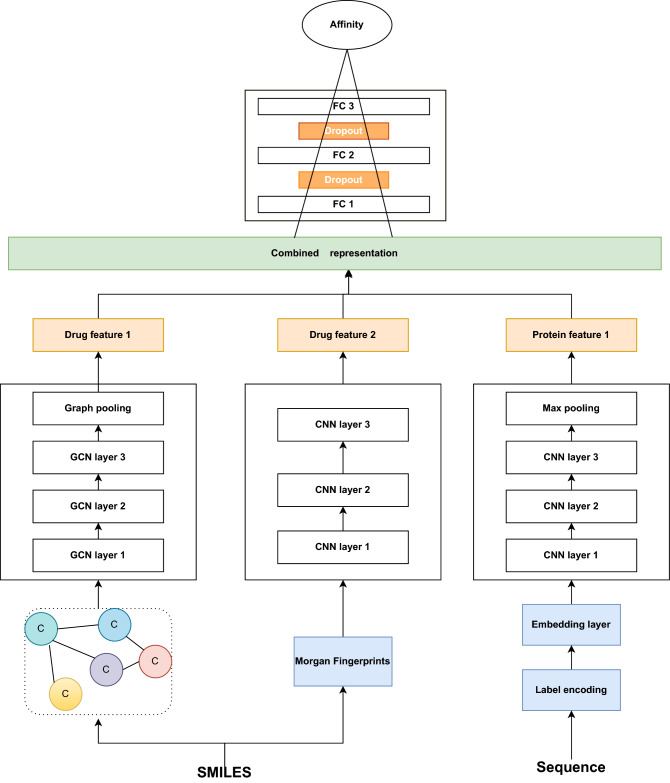
Detailed structure of the DeepDGC model.

The DeepDGC model (Fig. 2) was used in both processes P2 and P3, the input data types being SMILES sequences for compounds and protein amino acid sequences for targets. After compounds were transformed into SMILES strings and targets were converted into protein amino acid sequences, each SMILES string was matched to each amino acid sequence one by one. The output of this model was affinity values that indicated the interaction probabilities.

CNN was an architecture containing one or more convolutional layers, usually followed by a pooling layer, and GCN, an optimization of CNN, was a graph neural network using convolutional operations and compensated for the inability of CNN to handle non-Euclidean structured data. Therefore, the model combined GCN and CNN. It comprised two separate CNN blocks and a GCN block. For CNN block, we used DeepDTA’s^18^ configurations to set up the CNN block for the DeepDGC model, and for GCN block, we used three graph convolutional layers. In each layer, the covalent bonds and node information were extracted according to the molecular map [Highlight the innovative nature of DL model].

The KIBA dataset (pretraining dataset) for DeepDGC comprised primarily of the SMILES strings of 2111 active compounds, the amino acid sequences of 229 targets, and 118,254 binding affinity values. And it was employed to train the model using five-fold-cross-validation. Furthermore, to ensure the generalization ability, we set the activation functions relu and dropout. Finally, CI and MSE were used as evaluation indicators and the average results were reported. And with regard to the parameter settings, some of them were based on relevant studies, while other important parameters were compared experimentally for optimum results. The relevant parameters were set as shown in Table 1.

**Table 1:** The DeepDGC model hyperparameter settings.

Hyperparameters	Value range	Meaning of parameters
Batch_size	128	Number of samples in a single training session
Epoch	2000	Number of iterations
Activation function	ReLu	Activation function
Optimizer	Adam	Optimizer
Dropout	0.1	Random deactivation rate
Learning rate (lr)	1e-4	Learning rate

Subsequent to the evaluation of the pre-training dataset, the DeepDGC model was used to predict affinity values in process P2 and P3. The prediction dataset used in process P2 was composed primarily of the SMILES strings of the key compounds of licorice (obtained from the PPI analysis), and amino acid sequences of the key targets (obtained from the PPI analysis); while the prediction dataset used in process P3 was composed primarily of the SMILES strings of active compounds of licorice (screened by OB ≥ 30% and DL ≥ 0.18), and amino acid sequences of targets (for COVID-19).

### Selection of compliant core compounds by ADMET analysis

ADMET prediction is the assessment of five aspects (absorption, distribution, metabolism, excretion, and toxicity), which plays a key role in drug development^30^. In this study, the physicochemical and pharmacokinetic properties of the active components were predicted through the SwissADME database^31^ and the pkCSM database^32^, respectively.

The physicochemical properties considered here include molecular weight (MW), rotatable bond count (RB), H-bond acceptors (HBA), donor count, TPSA, and leadlikeness violations (LSV). The pharmacokinetic properties included absorption (i.e. Caco-2 cell permeability, HIA and skin permeability), distribution (i.e. VDss, unbound fraction, the blood–brain barrier and central nervous system permeability), excretion (i.e. total clearance and renal OCT2 substrate), and toxicity (i.e. AMES toxicity, maximum tolerated dose, hERG I inhibitor, hERG II inhibitor, oral rat acute toxicity (LD50), hepatotoxicity, skin sensitization, and minnow toxicity).

### Validation of core compounds and core targets by molecular docking

Molecular docking was used to further verify the binding capabilities of the core compounds and related targets outlined above. First, the monomeric component structures of the protein targets and related information were obtained from the UniProb^33^ and PDB^34^ databases. Second, AutoDockTools was used to conduct a range of operations, such as hydrogenation, charge addition, removal of water molecules, and removal of metal ions. Third, the PubChem database^35^ was used to construct the 3D structures of the active compounds. Subsequently, global docking boxes were generated by AutoDockTools while blind docking was performed using qvina-w. The binding score was used to evaluate the ability of a natural compound to bind to the target. Finally, heat maps and 3D docking maps of the docking results were created using Python and Pymol.

### Molecular dynamics simulations

The compound-protein target pair with the highest binding energy of molecular docking was subjected to molecular dynamics (MD) simulations to further check the binding stability of the two. MD simulations were then performed using Gromacs software^36^. To ensure the total charge neutrality of the simulated systems, corresponding amounts of sodium ions were added to the three systems to displace water molecules and produce solvent boxes of appropriate size. Next, periodic boundary conditions (PBC) were applied in each of the three directions of the system, thereby determining the force field parameters for the entire atom. Finally, two complexes were simulated for a 100 ns NPT ensemble (with constant number of particles, pressure and temperature).

## Results

### Key compounds and key targets

To explore the mechanism underlying the therapeutic effects of licorice against COVID-19, 774 targets were imported into the STRING database to construct a PPI network. After screening according to three thresholds, we obtained 88 key compounds (Table S1) and 156 key targets (Table S2).

### Core compounds and core targets

**Figure 3:**
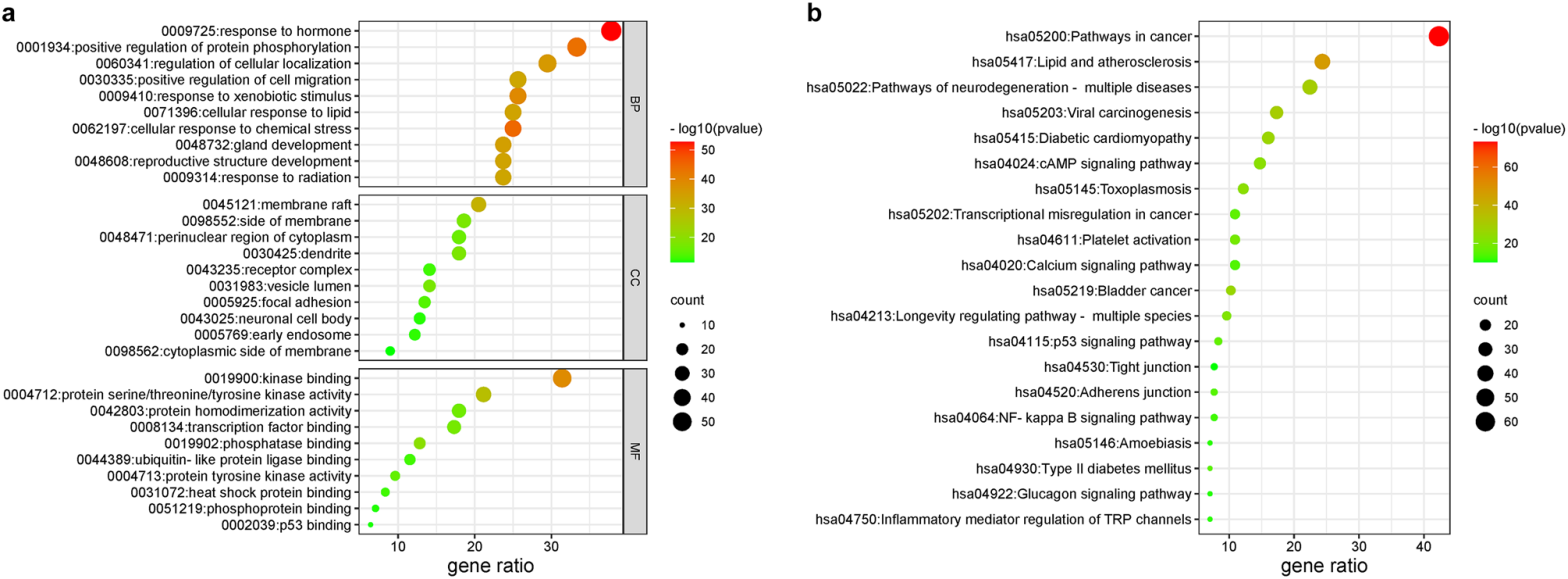
Enrichment analysis of GO and KEGG. (**a**) Top 10 biological processes, top 10 cellular compounds, and top 10 molecular functions: (**b**) top 20 KEGG pathways. The color scale indicates the different thresholds of *p* values and the size of the dots represents the number of genes corresponding to each pathway.

To determine the molecular mechanisms underlying the efficacy of licorice treatment against COVID-19, we used Metascape to perform the biofunctional annotation of GO and KEGG pathway enrichment analysis of the key targets. The GO biofunctional annotation results showed that a variety of terms were identified, including 254 biological process (BP) terms that mainly relate to the positive regulation of protein phosphorylation and the positive regulation of cell migration, 141 cellular component (CC) terms that mainly relate to the lumen of the vesicle, membrane rafts, and receptor complexes, 59 molecular function (MF) terms that were mainly related to kinase binding, protein serine/threonine/tyrosine kinase activity and transcription factor binding. The top 10 considerably enriched terms for BP, CC, and MF were visualized in Fig. 3a.

The KEGG pathway enrichment analysis results showed that the key targets were enriched in 180 pathways, and the top 20 paths with the highest level of enrichment were chosen for visualization in Fig. 3b, which revealed enrichment mainly in the P53 signaling pathway, cAMP signaling pathway, NF-kB, and other related signaling pathways.

Furthermore, to better understand the mechanism by which licorice acts on COVID-19, a licorice compound-target-pathway relationship network was built based on the above KEGG pathway (Fig. S1), which suggests the potential interactions between active compounds and targets, as well as the related pathways of licorice for the treatment of COVID-19. Finally, the top 5 active compounds (quercetin, glypallichalcone, calycosin, vestitol, naringenin) were selected as the core compounds and the top 5 genes (PTGS2, HSP90AB1, PPARG, SYK, ALB) were selected as the core targets. These results derived from process P1.

### Complementary core compounds and core targets

To explore more core compounds and core targets, a DeepDGC model was presented in this study. After the model was trained on the pre-training dataset, it was used to predict the affinity values between compounds and targets. We used four models (KronRLS^37^, SimBoost^38^, DeepDTA^15^, WideDTA^39^, DeepPurpose^19^ as baseline models and the comparison results were shown in Table 2.

**Table 2:** Performances of various methods on KIBA dataset.

Method	Proteins and compounds	MSE	CI
KronRLS^37^	S–W & Pubchem Sim	0.411	0.782
SimBoost^38^	S–W & Pubchem Sim	0.222	0.836
DeepDTA^15^	S–W & Pubchem Sim	0.502	0.710
DeepDTA^15^	CNN&Pubchem Sim	0.271	0.718
DeepDTA^15^	S–W &CNN	0.204	0.854
DeepDTA^15^	CNN & CNN	0.194	0.863
WideDTA^39^	PS + PDM & LS + LMCS	0.179	0.875
DeepPurpose^19^	GCN & CNN	0.177	0.879
DeepDGC	CNN & CNN + GCN	0.162	0.888

The prediction data set used in the process P2 was primarily composed of 88 SMILES strings of active compounds and 1248 amino acid sequences of targets. The prediction data set used in the P3 process was composed primarily of 92 SMILES strings of active compounds and 58,378 amino acid sequences of targets. The final prediction affinity values of the top 30 for P2 and P3 were shown in Tables 3 and 4.

**Table 3:** Top 30 drug-target pairs obtained by the P2 process.

Key target	Molecule name	Mol ID	Affinity	Key target	Molecule name	Mol ID	Affinity
JAK2	Shinflavanone	MOL004805	12.553	SYK	Naringenin	MOL004328	12.314
JAK2	Phaseolinisoflavan	MOL004833	12.524	SYK	Vestitol	MOL000500	12.310
PTEN	Xambioona	MOL005018	12.503	SYK	Calycosin	MOL000417	12.306
HSP90AB1	Vestitol	MOL000500	12.496	SYK	Glypallichalcone	MOL004835	12.305
IDH1	Glyasperin B	MOL004808	12.446	JAK2	Glyasperin B	MOL004808	12.292
PTEN	Phaseolinisoflavan	MOL004833	12.432	LYN	Gancaonin G	MOL005000	12.284
HSP90AB1	Calycosin	MOL000417	12.392	ERBB4	Inermine	MOL001484	12.265
PTEN	Glabrone	MOL004912	12.373	ERBB4	Glyasperin B	MOL004808	12.265
PTEN	Licoisoflavone B	MOL004884	12.373	ERBB4	Glyasperin C	MOL004811	12.263
HSP90AB1	Quercetin	MOL000098	12.363	LYN	Licoisoflavone B	MOL004884	12.252
HSP90AB1	Naringenin	MOL004328	12.359	IDH1	Shinflavanone	MOL004805	12.243
JAK2	Licoisoflavone	MOL004883	12.343	IDH1	Xambioona	MOL005018	12.237
PTEN	Licoisoflavone	MOL004883	12.338	IDH1	Licoisoflavone B	MOL004884	12.237
HSP90AB1	Glypallichalcone	MOL004835	12.326	SYK	Xambioona	MOL005018	12.223
SYK	Quercetin	MOL000098	12.315	IDH1	Glabrone	MOL004912	12.219

**Table 4:** Top 30 drug-target pairs obtained by the P3 process.

Key target	Molecule name	Mol ID	Affinity	Key target	Molecule name	Mol ID	Affinity
RET	Licoisoflavone B	MOL004884	12.259	KIT	Semilicoisoflavone B	MOL004827	12.125
MAP3K8	Glabrene	MOL004911	12.218	RET	Glyasperin B	MOL004808	12.119
RET	Glabrene	MOL004911	12.207	MAP3K8	Semilicoisoflavone B	MOL004827	12.113
RET	Isotrifoliol	MOL004814	12.193	SYK	Vestitol	MOL000500	12.113
RET	Semilicoisoflavone B	MOL004827	12.189	MAP3K8	Lupiwighteone	MOL003656	12.109
MAP3K8	Calycosin	MOL000417	12.181	SYK	Calycosin	MOL000417	12.106
RET	Kaempferol	MOL000422	12.179	FGG	Semilicoisoflavone B	MOL004827	12.103
RET	Calycosin	MOL000417	12.165	MAP3K8	Licoisoflavone B	MOL004884	12.099
RET	Liquiritin	MOL004903	12.160	SYK	Glypallichalcone	MOL004835	12.089
MAP3K8	Glypallichalcone	MOL004835	12.154	MAP3K8	Liquiritin	MOL004903	12.077
RET	Glypallichalcone	MOL004835	12.149	MAP3K8	Glyasperin B	MOL004808	12.066
RET	Liquiritigenin	MOL001792	12.149	FGG	Licoisoflavone B	MOL004884	12.057
RET	Lupiwighteone	MOL003656	12.147	KIT	Licoisoflavone B	MOL004884	12.050
SYK	Quercetin	MOL000098	12.131	FLT1	Liquiritin	MOL004903	12.024
SYK	Naringenin	MOL004328	12.125	FLT3	Liquiritigenin	MOL001792	12.020

The combined results of the three processes (P1, P2 and P3), including all core compounds and core targets, were shown in Table 5. Compared with existing studies of formulations or formulae containing licorice, the relevance of the 4 compounds (quercetin^40^, naringenin^40^, liquiritigenin^41^ and licoisoflavanone^42^) and 6 targets (SYK^41^, PTGS2^43^, PPARG^43^, ALB^43^, HSP90AB1^44^ and JAK2^45^) had been explored previously. Therefore, the other 15 core compounds (glypallichalcone, calycosin and vestitol) and 2 core targets (PTEN and MAP3K8) will be discussed below.

**Table 5:** Summary of core compounds and core targets.

Process	Core compounds	Core targets
P1	Quercetin, glypallichalcone, calycosin, vestitol, naringenin	PTGS2, HSP90AB1, PPARG, SYK, ALB
P2	Shinflavanone, phaseolinisoflavan, xambioona, glyasperin B, calycosin, glabrone, licoisoflavone B, quercetin, naringenin licoisoflavone, glypallichalcone, gancaonin G, inermine, glyasperin C	HSP90AB1, SYK, JAK2, PTEN
P3	Licoisoflavone B, glabrene, isotrifoliol, semilicoisoflavone B, calycosin, kaempferol, liquiritin, glypallichalcone, liquiritigenin, lupiwighteone, quercetin, naringenin, glyasperin B, vestitol lupiwighteone, quercetin, naringenin, glyasperin B	SYK, RET, MAP3K8

### Non-toxic and easily absorbed core compounds

Based on the above results, ADMET was used to predict the physicochemical and pharmacokinetic properties according to SwissADME and pkCSM. SwissADME calculations showed that 5 compounds passed the stringent lead-like criteria (250 g/mol ≤ MW ≤ 350 g/mol, XLOGP ≤ 3.5 and rotatable bonds ≤ 7^46^), indicating they could be considered excellent drug candidates against COVID-19 (Table 6). These lead-like compounds were further predicted by pkCSM. Regarding absorption parameters and drug distribution parameters, all 5 compounds were within the acceptable range. However, isotrifoliol, glypallichalcone and calycosin did not satisfy the criteria of hERG II inhibitor. Finally, only the new active compounds glabrone (MOL004912) and vestitol (MOL000500) could be considered eligible core compounds, of which vestitol was identified by P1, and glabrone was identified in P2 and P3 (Table 7).

**Table 6:** Lead-like compounds.

Mol ID	MW	Rotatable bonds	H-Bond acceptors	H-Bond donors	TPSA	XLOGP	GI Absorption	Lipinski violations
MOL004814	298.25	1	6	2	93.04	2.74	High	0
MOL004912	336.34	1	5	2	79.90	3.39	High	0
MOL004835	284.31	5	4	1	55.76	3.28	High	0
MOL000417	284.26	2	5	2	79.90	2.44	High	0
MOL000500	272.30	2	4	2	58.92	2.94	High	0

**Table 7:** Non-toxic compounds.

Mol ID	Absorption			Distribution				Excretion		Toxicity							
	Caco2	HIA	Skin	VDss	FU	BBB	CNS	TC	OCT	AMES	MTDD	hERG I	hERG II	LD50	HT	SS	MT
MOL004814	0.36	96.24	− 2.74	− 0.16	0.09	− 0.36	− 2.19	0.75	No	Yes	0.28	No	Yes	2.38	No	No	0.02
MOL004912	0.70	92.64	− 2.78	0.07	0.09	− 0.31	− 1.76	0.44	No	No	− 0.22	No	No	2.13	No	No	0.64
MOL004835	1.32	93.48	− 2.81	− 0.20	0.07	− 0.34	− 2.21	0.74	No	Yes	0.56	No	Yes	2.09	No	No	0.87
MOL000417	1.08	94.51	− 2.75	0.06	0.08	− 0.07	− 2.20	0.20	No	No	0.06	No	Yes	2.35	No	No	0.19
MOL000500	1.19	93.07	− 2.88	0.34	0.09	− 0.07	− 2.14	0.31	No	No	− 0.50	No	No	2.35	No	No	0.69

### Stable combination of core compounds and core targets

**Figure 4:**
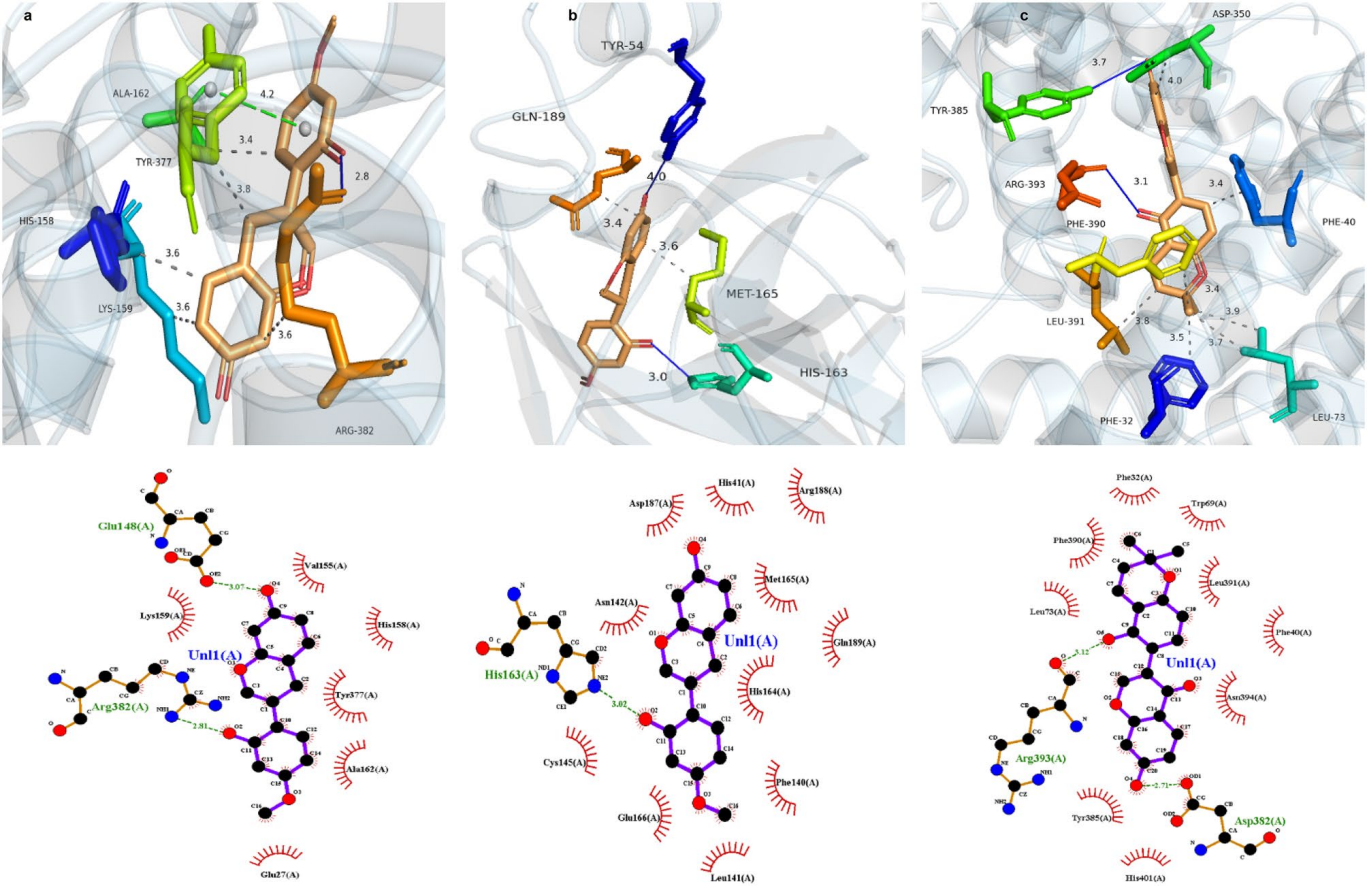
Molecular docking analysis of the selected compounds and targets with the largest binding values. (**a**) The docking mode of glabrone and S-protein. (**b**) The docking mode of glabrone and Mpro. (**c**) The docking mode of vestitol and ACE2.

To support our findings mentioned above, we used molecular docking to evaluate the interaction between the active core compounds and the core targets, in which binding affinity less than − 7.0 kcal/mol indicated a good interaction^47^. Two new active compounds in licorice (glabrone and vestitol) were docked to COVID-19 binding sites, such as Mpro, S-protein and ACE2. The binding affinities were shown in Table 8, and the binding modes of the selected active compounds and targets with the highest binding values was shown in Fig. 4. According to Tables 3 and 4, two new targets in COVID-19 (PTEN and MAP3K8) were docked to licorice-related compounds, such as glabrone, licoisoflavone B and isotrifoliol. The binding affinity results were shown in Table S3.

**Table 8:** Molecular docking results for glabrone and vestitol.

Key target	Target structure ID	Molecule name	Mol ID	PubChum Cid	Bingding score (kcal/mol)
S-protein	7kce	Glabrone	MOL004912	5,317,652	− 9.3
Mpro	7ng3	Glabrone	MOL004912	5,317,652	− 7.6
ACE2	7u0n	Glabrone	MOL004912	5,317,652	− 8.8
S-protein	7kce	Vestitol	MOL000500	92,503	− 7.5
Mpro	7ng3	Vestitol	MOL000500	92,503	− 7.5
ACE2	7u0n	Vestitol	MOL000500	92,503	− 7.7

### Molecular dynamics simulations

**Figure 5:**
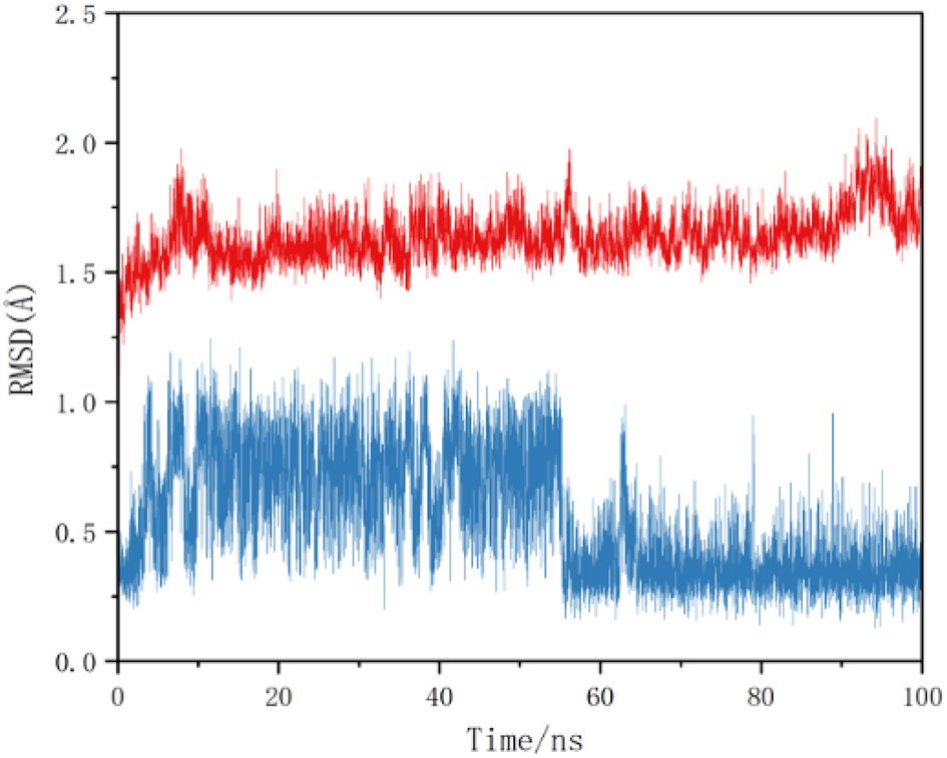
Differences in the root mean square deviation (RMSD) of the plural over time (blue polygonal line means glabrone and S-protein, red polygonal line means licoisoflavone B and MAP3K8).

The larger the protein Calpha root-mean-square deviation (RMSD) of the MD simulations, the more violent the fluctuations indicating greater motility and less stability.The RMSD data of licorice, include glabrone and S-protein, as well as licoisoflavone B and MAP3K8, were shown in Fig. 5. The results showed that the RMSD fluctuations for S-protein/glabrone and MAP3K8/licoisoflavone B are within 2 Å, which means that the system is less kinetic and more stable. These findings all showed that a stable conformation has been achieved in the process of MD simulations [MD simulations analysis].

## Discussion

The study design was divided into three processes, including the traditional network pharmacology process (P1) and the two improved processes (P2 and P3). Using this approach 20 core compounds and 6 core targets were predicted, among which 4 active compounds (quercetin, naringenin, liquiritigenin and licoisoflavanone) and 2 targets (SYK and JAK2), were associated with COVID-19 infection, as confirmed by previous studies. Furthermore, after ADMET and molecular docking analysis, two new active compounds were identified: vestitol was identified in P1 and glabrone was jointly identified in processes P2 and P3. and 2 new targets were also predicted, among which PTEN was identified in P2, and MAP3K8 were identified in P3. In addition, this study also revealed the signaling pathways of P53, cAMP, and NF-kB.

Considering the 4 active compounds (quercetin, naringenin, liquiritigenin and licoisoflavanone), their potential roles in the treatment of COVID-19 have been demonstrated in previous studies. Quercetin has anti-inflammatory activity exerted by inhibiting the secretion of pro-inflammatory factors (such as IL-6, IL-1β and TNF-α), by an antiviral effect by blocking the entry of coronavirus into host cells, as well as by an anticoagulant activity exerted by inhibiting plasma protein disulfide isomerase^48^. Naringenin also induces anti-inflammatory and antiviral activity^49^. As a flavonoid with anticancer, antioxidant, hepatoprotective, immune regulatory, and antiplatelet aggregation properties, liquiritigenin has served as a therapy for COVID-19. For example, liquiritigenin was used to form a complex with the Mpro of SARS-CoV2 because it was found to inhibit the catalytic activity of the main protease^50^. Licoisoflavanone is an isoflavonoid compound that plays a role in the reduction of antiviral, cytokine storms, prevention of ARDS and multi-organ damage, and reduction of the severity of inflammatory diseases^51^.

Previous studies have also indicated that SYK and JAK2 may serve as target proteins related to COVID-19. SYK also plays an important role in the treatment of COVID-19 and has been reported to regulate signal transduction pathways implicated in these complications associated with COVID-19^52^. JAK2 involves in M2 macrophage polarisation, inflammatory response, pulmonary fibrosis and thrombosis by activating STAT3, a signal transduction and transcriptional activator^53^. An increasing number of studies have also highlighted that JAK2 is an important gene belonging to the JAK2/STAT3 signaling pathway, and can induce overexpression of IL-6 and IL-18, which can exacerbate the inflammatory response and lung injury^46^. Furthermore, the SYK inhibitor (fostamatinib)^54^ and the JAK2 inhibitor (fedratinib)^55^, evaluated in clinical studies, are highly effective in the prevention and treatment of COVID-19.

The examples given above demonstrate the important roles of the 4 active compounds and 2 targets in COVID-19, identified in our analysis, and confirmed by previous studies, suggesting the usefulness of our model in the prediction of active compounds and targets. In fact, we also identified 2 new compounds (vestitol and glabrone) and 2 new targets (PTEN and MAP3K8) that have not yet been demonstrated to be directly associated with the treatment of COVID-19, but have been attested to have a role in the treatment of COVID-19-related diseases (e.g. hepatitis B, influenza A virus).

Many related studies had shown that the newly identified active compounds vestitol and glabrone play a key role in the treatment of COVID-19. Because they not only inhibit viral replication through stable binding to the three viral binding sites of S-protein, ACE2, and Mpro, but also exert antioxidant, anti-inflammatory, and antiviral effects. For example, vestitol can achieve an anti-inflammatory effect by inhibiting the NF-kB signaling pathway and has shown to be a considerable promising new anti-inflammatory agent^56^. Glabrone can achieve an antioxidant activity by modulating the nuclear factor erythroid 2-related factor 2 (Nrf2) pathway and an anti-inflammatory effect by regulating the NF-kB signaling pathway^57^. In addition, the antiviral activity of glabrone was demonstrated by cytopathic effect (CPE) inhibition assays targeting the influenza A virus^58^.

Two new targets identified using our approach, including PTEN and MAP3K8, were potentially key targets for the treatment of COVID-19. Studies had shown that PTEN can activate dendritic cells, B cells and T cells, which are innate immune cells, and secrete pro-inflammatory factors, including interferon (IFN), TNF-α and IL10, thus inducing the formation of the cytokine storm in patients with COVID-19. Therefore, targeting PTEN can inhibit the formation of cytokines storms^59^. MAP3K8 participates in the pulmonary fibrotic response and the lung inflammatory response. An increasing number of studies had also highlighted the significance of MAP3K8 in suppressing lung inflammation and fibrosis (the main symptom of COVID-19)^60^.

In terms of the signaling pathway, the P53, cAMP, and NF-kB signaling pathways, discovered by using KEGG pathway analysis, are involved in inflammation, immunomodulation and infection. The P53 signaling pathway is a pathway known to influence immune responses^61^. Furthermore, p53, an intrinsic host restriction factor of SARS-CoV-2, can reduce virus production^62^. The cAMP signaling pathway is the most important signaling pathway in EG pathway enrichment, and EG could also act on the PI3K-Akt, JAK-STAT and chemokine signaling pathways, thus reducing responses such as inflammation and apoptosis^40^. In turn, the NF-kB signaling pathway, considered as an inflammation center^63^, induces various target genes in inflammatory diseases^64,65^, as well as regulates cytokine storm syndromes and immunosuppression^66,67^.

S-protein and ACE2 were key protein targets in the first process of infection (attachment and cell entry)^68,69^, and Mpro was a key target in the second process of infection (replication and transcription)^70^. In this paper, we preliminarily concluded that vestitol and glabrone had good binding stability with S-protein, ACE2 and Mpro by molecular docking. Analysis of the S protein and glabrone was then further performed by MD simulations to demonstrate that glabrone may inhibit host cell infection at the first stage of attachment and entry. In addition, we also conducted molecular docking of two new targets (PTEN and MAP3K8) with the compounds of licorice (including vestitol and glabrone), and the results showed good binding stability. We followed by MD simulations of MAP3K8 and licoisoflavone B with the highest binding energy, which demonstrated that licorice may be considered an effective candidate for the treatment of COVID-19 through MAP3K8 [MD simulations analysis].

Based on the above analysis, we knowed that the DL-based network pharmacology method could compensate to some extent for the impossibility of obtaining all compounds and targets in traditional network pharmacology through databases and analysis software, and achieve a promising predictive results. However, due to the predictive performance of DL was very dependent on the quantity and quality of the data, and it also had limitations in feature information extraction. Therefore, there was still room for improvement in future work in terms of the quantity and quality of data and feature extraction [Highlight the innovation of the method and the shortcomings of the new method].

## Conclusion

In this study, we proposed a DL-based DeepDGC model that learned from both molecular maps and Morgan fingerprint data representations of drugs, which contains more feature information for drug characterization that can be evaluated and optimize the model. As a result, 2 new compounds and 2 new targets were also found to possess potential effects on COVID-19 treatment. Although our findings are not sufficient to reach more definite conclusions and further validation, using in vivo or in vitro studies, is still encouraged, we believe that this method has a certain translational value that can also be applied to drug and target discovery studies in other diseases.

## Supplementary Information

Supplementary Information.

## Data Availability

The datasets for this study can be found in the https://github.com/2022-fuyu/COVID-19.
