# Supplementary material for: Deep learning-based network pharmacology for exploring the mechanism of licorice for the treatment of COVID-19
Source: Sci Rep. 2023 Apr 10;13:5844. doi: 10.1038/s41598-023-31380-7 (PMC10086012; doi:10.1038/s41598-023-31380-7)
Supplement: Supplementary file 1 — Supplementary Information. [file 41598_2023_31380_MOESM1_ESM.pdf]

# Deep learning-based network pharmacology for exploring the mechanism of licorice for the treatment of COVID-19

Yu Fu[1], Yangyue Fang[1], Shuai Gong[1], Tao Xue[1], Peng Wang[1], Li She[1], and Jianping Huang[1]

[1] Alibaba Business School, Hangzhou Normal University, Hangzhou/310000, China.

Correspondence should be addressed to

Jianping Huang; 13167638797@163.com.

**Table S1** key compounds of licorice

| Mol ID | Molecule Name | OB (%) | DL |
|---|---|---|---|
| **MOL001484** | Inermine | 75.18 | 0.54 |
| **MOL001792** | DFV | 32.76 | 0.18 |
| **MOL000211** | Mairin | 55.38 | 0.78 |
| **MOL002311** | Glycyrol | 90.78 | 0.67 |
| **MOL000239** | Jaranol | 50.83 | 0.29 |
| **MOL002565** | Medicarpin | 49.22 | 0.34 |
| **MOL000354** | isorhamnetin | 49.6 | 0.31 |
| **MOL000359** | sitosterol | 36.91 | 0.75 |
| **MOL003656** | Lupiwighteone | 51.64 | 0.37 |
| **MOL003896** | 7-Methoxy-2-methyl isoflavone | 42.56 | 0.2 |
| **MOL000392** | formononetin | 69.67 | 0.21 |
| **MOL000417** | Calycosin | 47.75 | 0.24 |
| **MOL000422** | kaempferol | 41.88 | 0.24 |
| **MOL004328** | naringenin | 59.29 | 0.21 |
| **MOL004805** | (2S)-2-[4-hydroxy-3-(3-methylbut-2-enyl)phenyl]-8,8-dimethyl-2,3-dihydropyrano[2,3-f]chromen-4-one | 31.79 | 0.72 |
| **MOL004806** | euchrenone | 30.29 | 0.57 |
| **MOL004808** | glyasperin B | 65.22 | 0.44 |
| **MOL004810** | glyasperin F | 75.84 | 0.54 |
| **MOL004811** | Glyasperin C | 45.56 | 0.4 |
| **MOL004814** | Isotrifoliol | 31.94 | 0.42 |
| **MOL004815** | (E)-1-(2,4-dihydroxyphenyl)-3-(2,2-dimethylchromen-6-yl)prop-2-en-1-one | 39.62 | 0.35 |
| **MOL004820** | kanzonols W | 50.48 | 0.52 |

| | | | |
|---|---|---|---|
| **MOL004824** | (2S)-6-(2,4-dihydroxyphenyl)-2-(2-hydroxypropan-2-yl)-4-methoxy-2,3-dihydrofuro[3,2-g]chromen-7-one | 60.25 | 0.63 |
| **MOL004827** | Semilicoisoflavone B | 48.78 | 0.55 |
| **MOL004828** | Glepidotin A | 44.72 | 0.35 |
| **MOL004829** | Glepidotin B | 64.46 | 0.34 |
| **MOL004833** | Phaseolinisoflavan | 32.01 | 0.45 |
| **MOL004835** | Glypallichalcone | 61.6 | 0.19 |
| **MOL004838** | 8-(6-hydroxy-2-benzofuranyl)-2,2-dimethyl-5-chromenol | 58.44 | 0.38 |
| **MOL004841** | Licochalcone B | 76.76 | 0.19 |
| **MOL004848** | licochalcone G | 49.25 | 0.32 |
| **MOL004849** | 3-(2,4-dihydroxyphenyl)-8-(1,1-dimethylprop-2-enyl)-7-hydroxy-5-methoxy-coumarin | 59.62 | 0.43 |
| **MOL004855** | Licoricone | 63.58 | 0.47 |
| **MOL004856** | Gancaonin A | 51.08 | 0.4 |
| **MOL004857** | Gancaonin B | 48.79 | 0.45 |
| **MOL004860** | licorice glycoside E | 32.89 | 0.27 |
| **MOL004863** | 3-(3,4-dihydroxyphenyl)-5,7-dihydroxy-8-(3-methylbut-2-enyl)chromone | 66.37 | 0.41 |
| **MOL004864** | 5,7-dihydroxy-3-(4-methoxyphenyl)-8-(3-methylbut-2-enyl)chromone | 30.49 | 0.41 |
| **MOL004866** | 2-(3,4-dihydroxyphenyl)-5,7-dihydroxy-6-(3-methylbut-2-enyl)chromone | 44.15 | 0.41 |
| **MOL004879** | Glycyrin | 52.61 | 0.47 |
| **MOL004882** | Licocoumarone | 33.21 | 0.36 |
| **MOL004883** | Licoisoflavone | 41.61 | 0.42 |
| **MOL004884** | Licoisoflavone B | 38.93 | 0.55 |
| **MOL004885** | licoisoflavanone | 52.47 | 0.54 |
| **MOL004891** | shinpterocarpin | 80.3 | 0.73 |
| **MOL004898** | (E)-3-[3,4-dihydroxy-5-(3-methylbut-2-enyl)phenyl]-1-(2,4-dihydroxyphenyl)prop-2-en-1-one | 46.27 | 0.31 |
| **MOL004903** | liquiritin | 65.69 | 0.74 |
| **MOL004904** | licopyranocoumarin | 80.36 | 0.65 |
| **MOL004905** | 3,22-Dihydroxy-11-oxo-delta(12)-oleanene-27-alpha-methoxycarbonyl-29-oic acid | 34.32 | 0.55 |
| **MOL004907** | Glyzaglabrin | 61.07 | 0.35 |
| **MOL004908** | Glabridin | 53.25 | 0.47 |
| **MOL004910** | Glabranin | 52.9 | 0.31 |
| **MOL004911** | Glabrene | 46.27 | 0.44 |
| **MOL004912** | Glabrone | 52.51 | 0.5 |
| **MOL004913** | 1,3-dihydroxy-9-methoxy-6-benzofurano[3,2-c]chromenone | 48.14 | 0.43 |
| **MOL004914** | 1,3-dihydroxy-8,9-dimethoxy-6-benzofurano[3,2-c]chromenone | 62.9 | 0.53 |
| **MOL004915** | Eurycarpin A | 43.28 | 0.37 |

| | | | |
|---|---|---|---|
| **MOL004917** | glycyroside | 37.25 | 0.79 |
| **MOL004924** | (-)-Medicocarpin | 40.99 | 0.95 |
| **MOL004935** | Sigmoidin-B | 34.88 | 0.41 |
| **MOL004941** | (2R)-7-hydroxy-2-(4-hydroxyphenyl)chroman-4-one | 71.12 | 0.18 |
| **MOL004945** | (2S)-7-hydroxy-2-(4-hydroxyphenyl)-8-(3-methylbut-2-enyl)chroman-4-one | 36.57 | 0.32 |
| **MOL004948** | Isoglycyrol | 44.7 | 0.84 |
| **MOL004949** | Isolicoflavonol | 45.17 | 0.42 |
| **MOL004957** | HMO | 38.37 | 0.21 |
| **MOL004959** | 1-Methoxyphaseollidin | 69.98 | 0.64 |
| **MOL004961** | Quercetin der. | 46.45 | 0.33 |
| **MOL004966** | 3'-Hydroxy-4'-O-Methylglabridin | 43.71 | 0.57 |
| **MOL000497** | licochalcone a | 40.79 | 0.29 |
| **MOL004974** | 3'-Methoxyglabridin | 46.16 | 0.57 |
| **MOL004978** | 2-[(3R)-8,8-dimethyl-3,4-dihydro-2H-pyrano[6,5-f]chromen-3-yl]-5-methoxyphenol | 36.21 | 0.52 |
| **MOL004980** | Inflacoumarin A | 39.71 | 0.33 |
| **MOL004985** | icos-5-enoic acid | 30.7 | 0.2 |
| **MOL004988** | Kanzonol F | 32.47 | 0.89 |
| **MOL004989** | 6-prenylated eriodictyol | 39.22 | 0.41 |
| **MOL004990** | 7,2',4'-trihydroxy□5-methoxy-3□arylcoumarin | 83.71 | 0.27 |
| **MOL004991** | 7-Acetoxy-2-methylisoflavone | 38.92 | 0.26 |
| **MOL004993** | 8-prenylated eriodictyol | 53.79 | 0.4 |
| **MOL004996** | gadelaidic acid | 30.7 | 0.2 |
| **MOL000500** | Vestitol | 74.66 | 0.21 |
| **MOL005000** | Gancaonin G | 60.44 | 0.39 |
| **MOL005001** | Gancaonin H | 50.1 | 0.78 |
| **MOL005003** | Licoagrocarpin | 58.81 | 0.58 |
| **MOL005007** | Glyasperins M | 72.67 | 0.59 |
| **MOL005008** | Glycyrrhiza flavonol A | 41.28 | 0.6 |
| **MOL005012** | Licoagroisoflavone | 57.28 | 0.49 |
| **MOL005013** | 18α-hydroxyglycyrrhetic acid | 41.16 | 0.71 |
| **MOL005016** | Odoratin | 49.95 | 0.3 |
| **MOL005017** | Phaseol | 78.77 | 0.58 |
| **MOL005018** | Xambioona | 54.85 | 0.87 |
| **MOL005020** | dehydroglyasperins C | 53.82 | 0.37 |
| **MOL000098** | quercetin | 46.43 | 0.28 |

**Table S2** key targets of licorice

| Betweenness | Closeness | Degree | Gene | Betweenness | Closeness | Degree | Gene |
|---|---|---|---|---|---|---|---|
| 1844.27935 | 6.05E-04 | 51 | DNM1 | 1533.093687 | 6.50E-04 | 93 | CCNA2 |
| 1656.979785 | 6.28E-04 | 55 | AGTR1 | 3812.824564 | 6.74E-04 | 130 | ATM |
| 1778.888007 | 6.04E-04 | 61 | TPI1 | 5941.729217 | 6.92E-04 | 129 | APP |
| 8067.138406 | 6.97E-04 | 135 | CAT | 6206.104687 | 7.10E-04 | 162 | FOS |
| 3821.503654 | 6.67E-04 | 94 | SOD1 | 1705.651422 | 6.58E-04 | 81 | SNCA |
| 1785.757305 | 6.27E-04 | 50 | AKR1B1 | 3503.203887 | 6.61E-04 | 94 | MAPT |
| 1643.995711 | 6.49E-04 | 66 | ABCB1 | 1173.804792 | 6.76E-04 | 111 | PTPN11 |
| 4375.372914 | 7.25E-04 | 194 | CCND1 | 2378.805498 | 6.45E-04 | 81 | ERBB4 |
| 1977.537001 | 6.60E-04 | 90 | NR3C1 | 1989.684578 | 6.66E-04 | 115 | FYN |
| 2541.358097 | 6.39E-04 | 59 | ABCG2 | 1100.001272 | 6.35E-04 | 90 | RAC1 |
| 10448.76452 | 7.63E-04 | 245 | MAPK3 | 2618.199154 | 6.75E-04 | 129 | STAT1 |
| 5697.05864 | 7.02E-04 | 165 | EP300 | 2168.434133 | 6.51E-04 | 96 | PARP1 |
| 1631.222758 | 6.79E-04 | 121 | KDR | 1223.262176 | 6.51E-04 | 86 | RUNX2 |
| 8625.899488 | 7.58E-04 | 245 | STAT3 | 1177.325364 | 6.52E-04 | 76 | PTPN1 |
| 29379.73622 | 8.14E-04 | 319 | TP53 | 2116.072362 | 6.61E-04 | 108 | CDK1 |
| 5133.249163 | 7.23E-04 | 186 | ERBB2 | 1815.363239 | 6.76E-04 | 123 | JAK2 |
| 14698.3443 | 7.77E-04 | 263 | EGFR | 6165.189486 | 7.17E-04 | 177 | RHOA |
| 1095.970757 | 6.11E-04 | 48 | HMGCR | 1054.468741 | 6.18E-04 | 56 | MME |
| 1268.868217 | 6.61E-04 | 104 | KIT | 1954.0732 | 6.36E-04 | 67 | IDH1 |
| 5509.265518 | 6.92E-04 | 118 | HPGDS | 1333.740424 | 6.50E-04 | 104 | LYN |
| 33069.54863 | 8.14E-04 | 313 | ALB | 2129.198161 | 6.75E-04 | 136 | PIK3R1 |
| 1348.178663 | 6.88E-04 | 141 | ANXA5 | 6010.627825 | 6.84E-04 | 133 | HDAC1 |
| 2185.141193 | 6.91E-04 | 139 | BCL2L1 | 1897.573668 | 6.54E-04 | 83 | CDK5 |
| 6675.204712 | 7.54E-04 | 225 | CASP3 | 1874.984021 | 6.48E-04 | 79 | NTRK1 |
| 2047.085067 | 6.54E-04 | 88 | HNF4A | 2871.067666 | 6.81E-04 | 128 | GRB2 |
| 2205.255536 | 6.17E-04 | 63 | TYMS | 1225.708978 | 6.20E-04 | 51 | GCK |
| 12278.20379 | 7.65E-04 | 243 | HSP90AA1 | 1662.670494 | 6.38E-04 | 74 | PKM |
| 2377.601716 | 6.42E-04 | 68 | CYP3A4 | 1626.522034 | 6.31E-04 | 59 | G6PD |
| 5972.213059 | 7.10E-04 | 156 | CAV1 | 2015.97163 | 6.53E-04 | 90 | ADIPOQ |
| 1736.465955 | 6.49E-04 | 71 | HTT | 2761.294933 | 6.43E-04 | 72 | LDHA |
| 1568.009569 | 6.33E-04 | 60 | TOP1 | 3696.24583 | 6.70E-04 | 96 | ACE |
| 4511.843614 | 7.28E-04 | 184 | MTOR | 1533.262427 | 6.65E-04 | 94 | HMOX1 |
| 1619.429993 | 6.33E-04 | 61 | EZR | 1714.647846 | 6.81E-04 | 127 | MMP2 |

| | | | | | | | |
|---|---|---|---|---|---|---|---|
| 4441.689591 | 7.03E-04 | 154 | PTGS2 | 1472.429068 | 6.61E-04 | 97 | SERPINE1 |
| 1008.049131 | 6.46E-04 | 88 | XIAP | 2444.629062 | 6.38E-04 | 66 | CTSD |
| 2251.921788 | 6.92E-04 | 120 | HSP90AB1 | 1482.600085 | 6.57E-04 | 91 | CRP |
| 9000.691879 | 7.55E-04 | 230 | PTEN | 1835.534004 | 6.77E-04 | 130 | ICAM1 |
| 1494.259193 | 6.62E-04 | 107 | ABL1 | 2995.484315 | 6.52E-04 | 84 | REN |
| 13207.68454 | 7.73E-04 | 263 | SRC | 2268.121437 | 6.27E-04 | 58 | CALM1 |
| 1039.280819 | 6.84E-04 | 124 | MAPK8 | 2952.590367 | 6.58E-04 | 84 | LRRK2 |
| 1863.070561 | 6.40E-04 | 58 | GSTP1 | 7109.565387 | 7.17E-04 | 180 | CXCL8 |
| 1763.338922 | 6.74E-04 | 124 | RELA | 1904.63223 | 6.33E-04 | 55 | NOS1 |
| 2406.6149 | 6.35E-04 | 83 | SMARCA4 | 3135.122975 | 7.10E-04 | 163 | IGF1 |
| 2508.499623 | 6.14E-04 | 51 | DHFR | 2008.768015 | 6.50E-04 | 80 | F2 |
| 25909.07807 | 8.01E-04 | 302 | TNF | 5852.572371 | 7.18E-04 | 183 | MMP9 |
| 12018.89116 | 7.43E-04 | 217 | ESR1 | 1439.180893 | 6.57E-04 | 91 | PLG |
| 7766.573351 | 7.48E-04 | 220 | HRAS | 1561.061252 | 6.85E-04 | 123 | GSK3B |
| 2257.123841 | 6.55E-04 | 75 | SLC2A1 | 1905.761615 | 6.05E-04 | 59 | GRIN1 |
| 2193.618647 | 6.74E-04 | 98 | HSPA8 | 1396.850109 | 6.37E-04 | 71 | CTSB |
| 6791.577714 | 7.43E-04 | 212 | HIF1A | 1327.802829 | 6.40E-04 | 81 | TNFRSF1A |
| 30322.25521 | 8.38E-04 | 346 | AKT1 | 1253.388284 | 6.08E-04 | 47 | ARG1 |
| 9686.774837 | 7.66E-04 | 250 | VEGFA | 1262.447125 | 6.68E-04 | 94 | PRKCA |
| 1948.45357 | 6.28E-04 | 53 | DPP4 | 3063.822431 | 6.15E-04 | 56 | SERPINA1 |
| 1907.737474 | 6.32E-04 | 75 | APOB | 3070.179946 | 6.54E-04 | 84 | PRKACA |
| 12224.11742 | 7.22E-04 | 186 | PPARG | 1214.97581 | 6.56E-04 | 91 | AKT2 |
| 3730.435369 | 6.93E-04 | 129 | NOS3 | 2470.585896 | 6.73E-04 | 124 | IL2 |
| 2067.728225 | 6.44E-04 | 72 | FASN | 1061.908484 | 6.50E-04 | 68 | SIRT3 |
| 3036.236239 | 6.54E-04 | 89 | SREBF1 | 1605.12072 | 6.33E-04 | 55 | CYP19A1 |
| 6613.254923 | 6.84E-04 | 127 | PPARA | 3444.0957 | 6.07E-04 | 60 | GART |
| 1351.730728 | 6.11E-04 | 56 | LDLR | 1047.443653 | 6.51E-04 | 92 | RAF1 |
| 5074.311752 | 6.35E-04 | 61 | CFTR | 1409.314579 | 6.29E-04 | 49 | RAB5A |
| 3754.361753 | 6.82E-04 | 128 | AR | 1393.135354 | 6.21E-04 | 57 | PGK1 |
| 2788.00114 | 6.62E-04 | 91 | PGR | 2178.586658 | 6.53E-04 | 73 | ESR2 |
| 3724.961866 | 6.96E-04 | 144 | CDC42 | 2127.179252 | 6.22E-04 | 64 | NCOR2 |
| 1478.845441 | 6.43E-04 | 64 | GJA1 | 1210.678349 | 6.28E-04 | 54 | VCP |
| 1688.629094 | 6.70E-04 | 115 | ITGB1 | 1940.252634 | 6.77E-04 | 105 | HSPA5 |
| 1365.66376 | 6.43E-04 | 83 | MET | 2424.37837 | 6.62E-04 | 82 | SOD2 |
| 2685.143977 | 6.61E-04 | 89 | SLC2A4 | 1064.196738 | 6.54E-04 | 95 | CCNB1 |
| 1122.3942 | 6.34E-04 | 55 | AHR | 2471.388208 | 6.21E-04 | 58 | CSF1R |

| | | | | | | | |
|---|---|---|---|---|---|---|---|
| 3067.545736 | 6.81E-04 | 128 | CXCR4 | 1499.339768 | 6.75E-04 | 114 | MAP2K1 |
| 5565.786149 | 7.09E-04 | 162 | SIRT1 | 1012.770576 | 6.42E-04 | 63 | NOS2 |
| 7218.297667 | 7.26E-04 | 188 | MAPK1 | 2106.122208 | 6.20E-04 | 64 | PPP1CC |
| 1604.561419 | 6.46E-04 | 84 | MPO | 1366.320834 | 6.08E-04 | 49 | GRIN2A |
| 3394.538737 | 6.98E-04 | 145 | MAPK14 | 2506.982509 | 6.24E-04 | 61 | EIF4E |
| 1278.135215 | 6.65E-04 | 105 | CDK4 | 2241.573507 | 6.06E-04 | 51 | GRIA1 |
| 2290.055135 | 6.89E-04 | 138 | MDM2 | 1742.251525 | 6.49E-04 | 85 | HDAC2 |
| 2418.582787 | 6.82E-04 | 149 | PIK3CA | 3046.950241 | 6.24E-04 | 73 | GRIN2B |
| 1172.684076 | 6.67E-04 | 104 | CDK2 | 1071.113087 | 6.51E-04 | 89 | SYK |

**Table S3** Molecular docking results for new target

| Key Target | Target Structure ID | Molecule Name | Mol ID | Bingding Score (kcal/mol) |
|---|---|---|---|---|
| **MAP3K8** | 4y85 | MOL004912 | 480774 | -10 |
| **MAP3K8** | 4y85 | MOL000417 | 5280448 | -9.8 |
| **MAP3K8** | 4y85 | MOL004835 | 5317768 | -9.2 |
| **MAP3K8** | 4y85 | MOL004827 | 5481948 | -8.2 |
| **MAP3K8** | 4y85 | MOL003656 | 5317480 | -7.9 |
| **MAP3K8** | 4y85 | MOL004884 | 5481234 | -7.7 |
| **MAP3K8** | 4y85 | MOL004903 | 503737 | -7.5 |
| **MAP3K8** | 4y85 | MOL004808 | 480784 | -7 |
| **PTEN** | 7jul | MOL005018 | 14769500 | -9.8 |
| **PTEN** | 7jul | MOL004808 | 480784 | -9.4 |

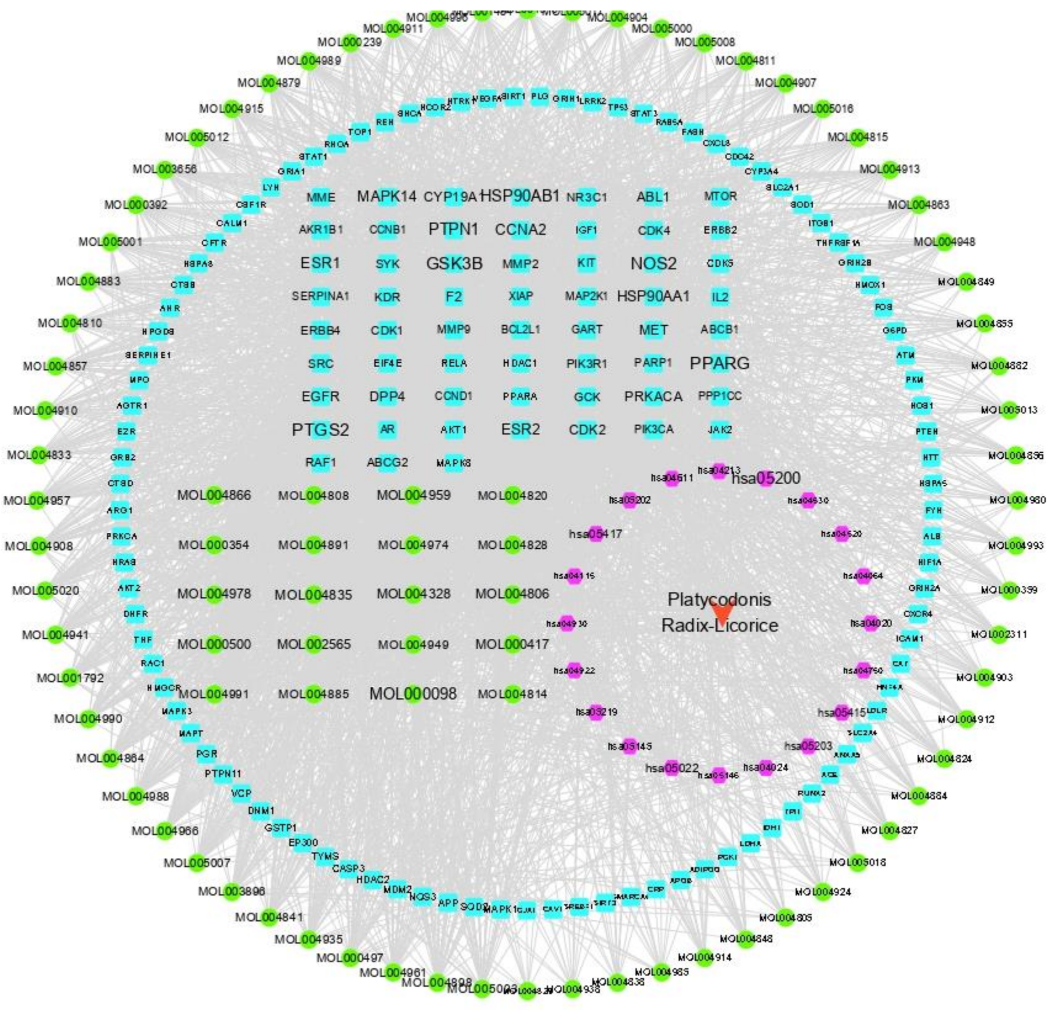


**Figure S1.** Licorice-compound-target-pathway network of licorice. The red V represent licorice. The green circles represent the active compounds of licorice. The blue squares represent the targets. The rose hexagons represent pathways. the larger fonts the greater degree value.
